# Vancomycin-Resistant *Enterococcus faecium* Sterilization and Conductivity Change by Impulse Voltage

**DOI:** 10.3390/microorganisms11020517

**Published:** 2023-02-17

**Authors:** Takahisa Ueno, Takashi Furukawa, Takashi Sakugawa

**Affiliations:** 1 Department of Electrical and Electronic Engineering, National Institute of Technology, Oita College, 1666 Maki, Oita-shi 870-0152, Oita, Japan; 2 Department of Health Science, School of Allied Health Sciences, Kitasato University, A1-505, 1-15-1 Kitasato, Minami-Ku, Sagamihara-shi 252-0373, Kanagawa, Japan; 3 Institute of Industrial Nanomaterials, Kumamoto University, Kurokami 2-39-1, Chuo-Ku, Kumamoto-shi 860-8555, Kumamoto, Japan

**Keywords:** conductivity, drug-resistant bacteria, impulse voltage, silicon-insulated gate bipolar transistor processes, sterilization, vancomycin

## Abstract

Owing to the increased use of antibiotics, drug-resistant strains, including those that are resistant to the antibiotic vancomycin, have emerged, which has become a major problem. In Japan, sewage treatments consist of sterilization with chlorine; however, this may not be sufficient to inactivate these bacteria. In this study, impulse voltage was employed instead of chlorine to inactivate drug-resistant bacteria. The results showed that sterilization above 10^5^ CFU/mL is possible with longer application times of applied voltages above 4.5 kV. The effectiveness of impulse-voltage-mediated sterilization increased as the temperature of the bacterial suspension increased. The number of bacteria sterilized via impulse voltage was correlated with conductivity when the number of bacteria sterilized by impulse voltage exceeded 10^5^ CFU/mL. The sterilization rate achieved by the use of impulse voltage could be estimated immediately by measuring the electrical conductivity and without the need for using the culture method.

## 1. Introduction

*Enterococcus* belongs to a genus of commonly found bacteria with low pathogenicity and is relatively harmless when detected in healthy individuals. In people with reduced immune function, *Enterococcus* can lead to serious infectious diseases, such as endocarditis or septicemia, which require treatment with antibiotics [1,2,3]. However, increased antibiotic use has resulted in the emergence of drug-resistant strains, including those resistant to vancomycin, which has become a major problem. In sewage treatment plants in Japan, the currently used concentration of chlorine may not completely sterilize drug-resistant bacteria [4]. This may lead to the propagation of resistance genes, thereby leading to the appearance of new strains of multidrug-resistant bacteria. Sterilization via ultraviolet irradiation and ozone are alternatives to chlorination; however, the required equipment is costly. Furthermore, the effectiveness of these methods is attenuated by turbidity, indicating that sufficient sterilization may not always be achieved.

In the present study, we proposed the use of impulse voltage as an alternative method of sterilization [5]. This technique uses an electric field to physically disrupt bacterial cell membranes [6,7], thereby inactivating the bacteria itself [8]. Since the experiments of Sale and Hamilton in 1967, a number of studies on impulse-voltage-mediated sterilization have been performed [9,10,11,12,13,14]. In the impulse voltage method, physical factors such as electric field and temperature distribution between electrodes, the orientation of bacteria to the electric field, and the nature of the medium as well as biological factors such as the cell cycle and membrane state affect the degree of membrane damage and response to damage. Until now, mostly bacteria that are likely to be detected in food, such as *E. coli* and *Enterobacter*, were subjected to sterilization by impulse voltage. To date, the authors have applied impulse voltage to vancomycin-resistant *Enterococcus faecium* (*VRE*) and achieved a reduction rate of >5 log. However, the temperature of the bacterial suspension was not controlled but increased by the energy of the impulse voltage [15].

In addition, since sterilization by impulse voltage causes intracellular fluid to flow out due to membrane damage, we predicted that intracellular ions might flow out and change the conductivity of the entire bacterial suspension. In order to determine the number of sterilized bacteria, it is necessary to know the number of viable bacteria. There are various methods for quantifying viable bacteria, such as the turbidity, plate culture, and polymerase chain reaction methods; however, these methods have disadvantages such as the time required for measurement and the high cost of the equipment. If the number of bacteria can be estimated by measuring conductivity, it may be possible to develop an inexpensive and simple method for quantifying bacteria, although its use is limited to sterilization by impulse voltage. Furthermore, the temperature was not constant but increased according to the number of times impulse voltage was applied. In this study, we clarified the sterilizing effect of impulse voltage on *VRE* when temperature was controlled, focused on the change in conductivity of the bacterial suspension after impulse voltage was applied, and investigated its relationship with the rate of sterilization.

## 2. Materials and Methods

### 2.1. Target Bacteria

*VRE* (Gram-positive bacterium) strain ATCC 51559, which possesses the *vanA* gene, was used as a representative antibiotic-resistant bacterium in this study. *VRE* is listed as a serious threat in the CDC publication “Antibiotic Resistance Threats in the United States” and is considered one of the most alarming threats to public health. *VRE* carries the *vanA* gene. These resistance genes are located on plasmids and can easily undergo horizontal gene transfer [16].

### 2.2. Design of an Impulse Voltage Generator

**Figure 1:**
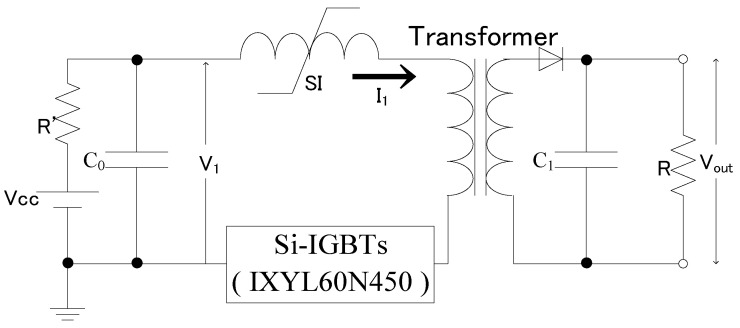
Impulse voltage generator circuit diagram. In this structure, the charged primary capacitor was discharged by a semiconductor, and the voltage was boosted using a transformer.

Figure 1 shows the circuit diagram of the impulse voltage generator that uses a transformer. The circuit configuration consists of a primary capacitor (*C*_0_ = 1.67 µF), SI (saturable inductor) device, transformer, and 10 silicon-insulated gate bipolar transistor processes (Si-IGBTs; IXYS, IXYL60N447) connected in parallel. The output voltage (*V_out_*) was applied to the load resistor (*R*) connected in parallel with the capacitor (*C*_1_ = 25 µF). The Si-IGBT is a discrete type of device with a rated voltage (*V_CES_*) of 4.5 kV and an instantaneous maximum current (<1 ms; I_CM_) of 680 A.

SI, which is comprised of windings on the iron core, was used to connect the current to the IGBTs in a parallel and uniform manner. In the current procedure, switching was controlled via an inductance change when the iron core was saturated and unsaturated; this is also known as a magnetic switch or saturable inductor. Therefore, permeability at saturation and unsaturation significantly affects the switching performance. Additionally, high-saturation magnetic flux density and slight core loss are required. In this section, the generation of *I*_1_ was delayed using SI, and *I*_1_ flowed after all Si-IGBTs connected in parallel were turned on, which enabled parallelization. The saturation time t_s_ (in µs) when a voltage Va (in V) is applied to a magnetic material with a running cross-sectional area Ae (in mm^2^), saturation flux density Bs (in mT), and number of turns N are calculated using the following equation [17]:

(1) $$t_{s}=\frac{N\cdot \mathrm{Ae}\cdot \mathrm{Bs}}{\mathrm{Va}}\;\times 10^{-3}$$

From the above equation, the delay time is directly proportional to the saturation flux density and number of turns of the magnetic material and inversely proportional to the applied voltage. SI used in this study was an iron core made of iron-based nanocrystalline alloy (Hitachi Metals, FT-3H, Bs = 1.23 mT) with a cross-sectional area of 1983 mm^2^. The number of turns was set to 15. The primary and secondary windings in the transformer were routed at equal intervals to equalize the magnetic flux distribution in the transformer, prevent leakage inductance, and increase the coupling factor.

**Figure 2:**
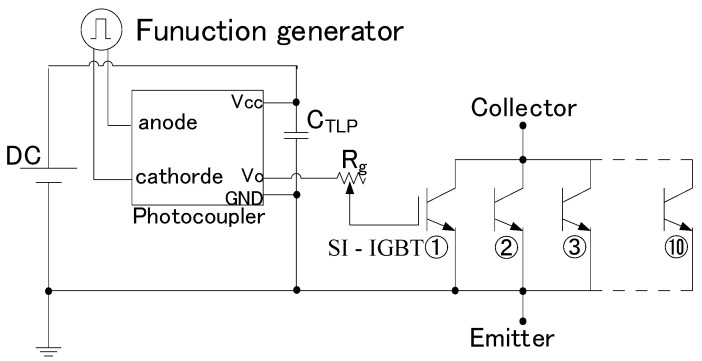
Si-IGBTs gate circuit diagram. A photocoupler was used to insulate the primary side from the secondary side. The gate signal output from the photocoupler was input to 10 IGBTs connected in parallel. The main current did not flow through the semiconductor in the current experiment considering that SI was unsaturated.

Figure 2 shows the gate circuit of the Si-IGBT. A pulse signal with a signal voltage of 5 V from a function generator was input to a photocoupler (Toshiba, TLP350(F)); the pulse signal was amplified to 22 V. The amplified pulse signal was transmitted to each Si-IGBT through the gate resistor *R_g_*, which was set to 8.0 Ω for the current experiment.

### 2.3. The Shape of Electrodes for Impulse Voltage Application

**Figure 3:**
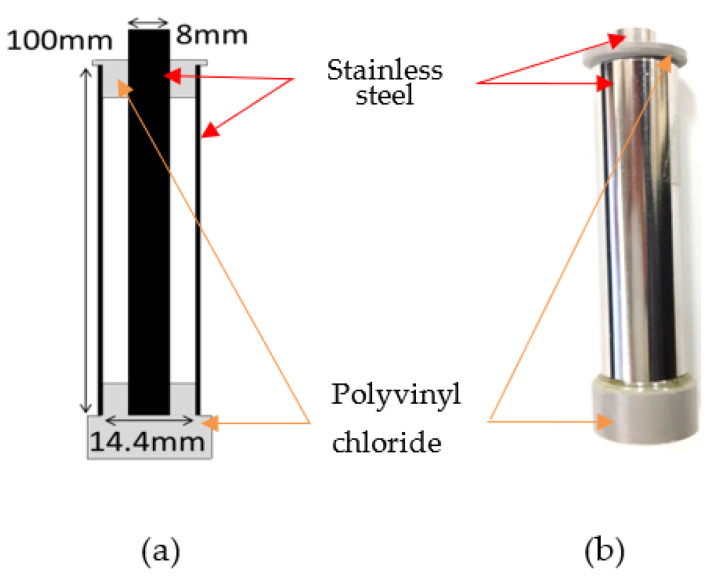
Cross-sectional diagram (**a**) and photograph (**b**) of the electrodes. The electrode had a coaxial cylindrical-type structure. Stainless steel was used as the conductor of the electrode, and vinyl chloride was used as the insulator. A pulse voltage was applied with the center conductor acting as the anode and the outer conductor as the cathode.

Figure 3 shows the shape of the electrodes used for bacterial and gene sterilization, including a cross-sectional diagram of the apparatus. The structure was that of a coaxial cylinder; stainless steel was used for the conductors, and polyvinyl chloride was used for the insulators. The inner conductor had a diameter of 8 mm, the outer conductor had a bore of 14.4 mm, and the space between the electrodes measured 3.2 mm. The apparatus was wired, with the inner conductor as the high-tension side and the outer conductor as the ground. The bacterial suspension was introduced into the space between the electrodes, and an impulse voltage was applied. The impulse voltage generator was developed using magnetic materials (Hitachi Metals, FT-3H, Bs = 1.23 mT) and semiconductor switches. The operating frequency was 200 Hz, and the impulse voltage was applied with a pulse width (full width at half maximum) of 6.9 µs or 1.7 µs.

### 2.4. Preparation of a Suspension and Bacterial Count

*VRE* at the late stage of logarithmic growth, i.e., when it is very active, were used for sterilization experiments where impulse voltage was applied. The optimum incubation time of 12–14 h was determined based on the *VRE* growth curve [15]. The strain was inoculated into Todd–Hewitt broth (Difco) supplemented with 128 µg/mL of vancomycin and incubated for 12–14 h at 37 °C ± 1.0 °C in a rotary shaker (120 rpm). Next, 5 mL of the precultured *VRE* strain was centrifuged at 4000× *g* for 5 min, and the supernatant was removed. The bacterial pellet was washed twice using 10 mL of sterile ultrapure water. The pellet was then suspended in 40 mL of sterile ultrapure water. The bacterial suspension’s absorbance was measured at 600 nm (OD_600_) using a spectrophotometer (AE-350, ERMA). Finally, the suspension containing *VRE* was adjusted to approximately 8.0 × 10^5^ CFU/mL and 8.0 × 10^3^ CFU/mL via dilution based on the calibration curve (OD_600_ absorbance vs. *VRE* count). The suspension was adjusted to approximately 8.0 × 10^9^ CFU/mL because the change in conductivity was small when the conductivity was measured. The suspension was stored at 25 °C ± 1.0 °C until use in the application experiment. The culturable *VRE* of each sample was counted using the spread plate method and Chromocult Enterococci Agar (Merck) plates. Samples predicted to have high bacterial concentrations were diluted with sterile phosphate-buffered saline; 100 µL of the sample or its prepared dilutions were inoculated on agar plates in triplicate and incubated at 35.0 °C ± 1.0 °C for 20–24 h. This was repeated five times for each condition. The sterilization rate (N) was expressed as the logarithm of the ratio of the number of bacteria (C_0_) before application to the number of bacteria (C) after application, as shown in Equation (2).

N = log_10_ (C_0_/C) (2)

### 2.5. Application of Impulse Voltage to the Bacterial Suspension

Impulse voltages with initial voltages of 0 kV, 1.5 kV, 4.5 kV, and 7.5 kV were applied to the bacterial suspension with an operating frequency of 200 Hz and a pulse width of 6.9 μs or 1.7 μs. The concentration of the suspension was 8.0 × 10^5^ CFU/mL and 8.0 × 10^3^ CFU/mL, and the application time was up to 10 min. The temperature change observed when the concentration of the suspension was 8.0 × 10^5^ CFU/mL was also measured.

### 2.6. Temperature Control of the Bacterial Suspension

Sterilization via application of impulse voltage was attempted after maintaining a constant temperature of the bacterial suspension inside the electrodes by cooling the outer conductor with circulating water whose temperature was controlled by a chiller. The concentration of bacteria was set to 5.0 × 10^5^ CFU/mL to clarify the effects of the impulse voltage versus the resulting increase in temperature. To clarify the relationship between electrical conductivity and bactericidal activity, a dilution of 8.0 × 10^9^ CFU/mL was also used. The temperature was adjusted to 20 °C, 40 °C, or 50 °C. The changes in the sterilization rate at an applied voltage of 7.5 kV, pulse width of 6.9 μs, and frequency of 200 Hz were measured via the colony method. In addition, the change in the sterilization rate without the impulse voltage was confirmed.

### 2.7. Conductivity Measurement of the Bacterial Suspension

Change in electrical conductivity when an impulse voltage was applied to a bacterial suspension of 8.0 × 10^9^ CFU/mL was measured. The conductivity was measured using a submersible electrical conductivity meter (Xylem, model 3200) at temperatures of 20 °C, 40 °C, and 50 °C of the bacterial suspension after application. Next, under the same bacterial concentration and electrical conditions mentioned in the previous section, an impulse voltage was applied to the bacterial suspension of *VRE*, and the conductivity was measured.

### 2.8. Statistical Analysis

Statistical analysis was conducted in Excel 2021 (Microsoft). To determine the relationships between each reduction ratio of *VRE* count and conductivity, with each electrical parameter (application time, voltage, and pulse width), the multiple regression analysis was carried out using *VRE* counts or conductivity as a response variable, and initial voltage, frequency, and pulse width as explanatory variables. The *p*-values were computed at a confidence level of 95%.

## 3. Results

### 3.1. Generation of the Primary-Side Current of the Impulse Voltage Generator

**Figure 4:**
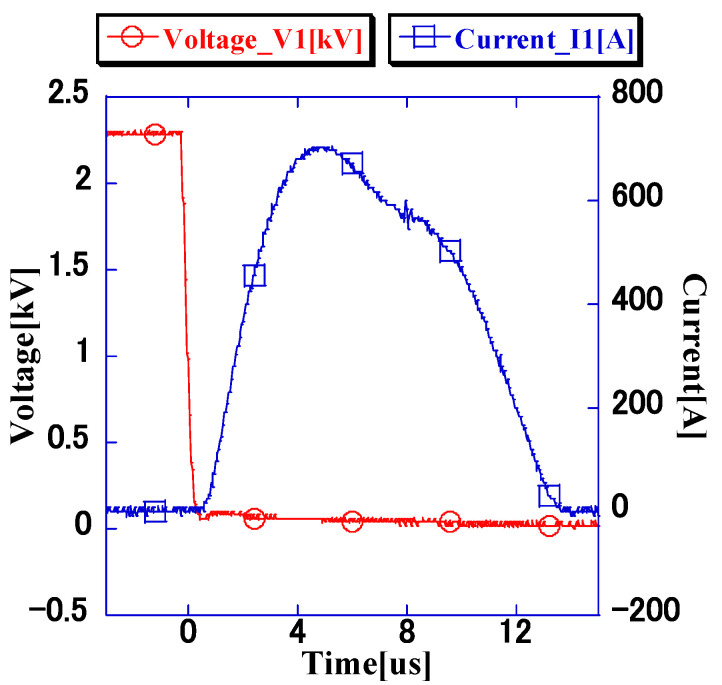
Voltage and current waveforms of the primary side. A current of approximately 728 A flowed through the semiconductor for 0.8 μs after the voltage applied to the semiconductor reduced.

Figure 4 shows the waveform of the primary-side voltage (*V*_1_ = 2.3 kV) and current for this configuration. It was observed that *I*_1_ = 728 A was flowing to the Si-IGBTs connected in parallel. The parallel connection of Si-IGBTs enabled the switching of the current, which exceeded the device’s rated current. The electrodes shown in Figure 1 were connected to the developed impulse voltage generator, and high impulse voltage was applied to the bacterial suspension.

### 3.2. Sterilization by Impulse Voltage with Slow Buildup

**Figure 5:**
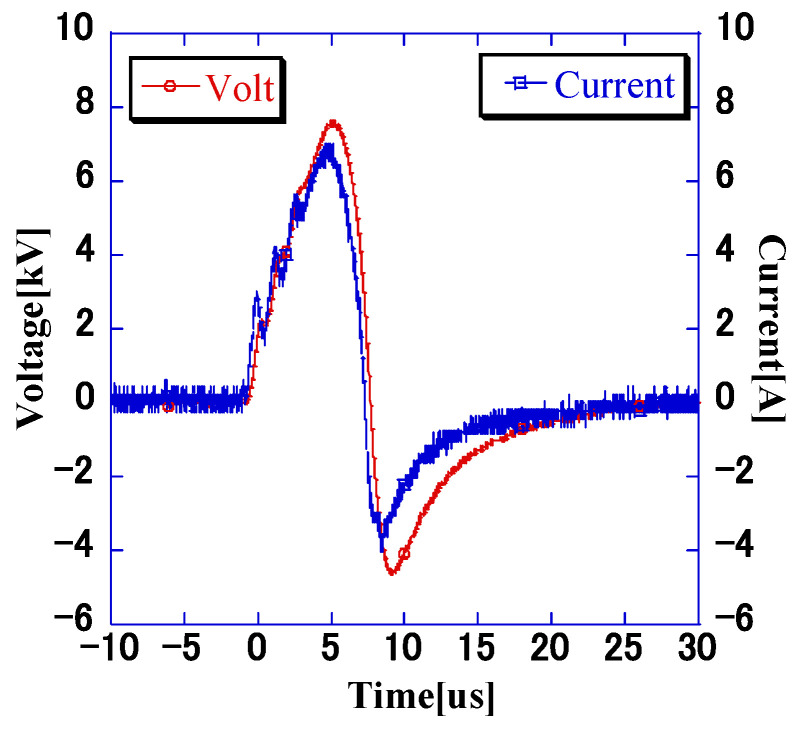
Voltage and current waveforms at a pulse width of 6.9 µs. The shape of the voltage and current waveforms are similar.

**Figure 6:**
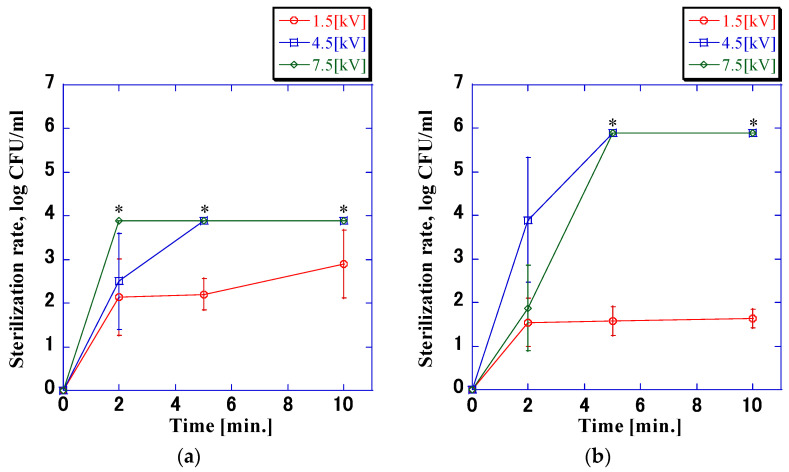
*VRE* sterilization rate at impulse voltage application times of up to 10 min (pulse width: 6.9 μs). The sterilization rate increased with the application time. In addition, there was a change in the sterilization ratio between the applied voltages 4.5 kV and 1.5 kV. There was no difference between the sterilization rates of the initial concentrations. Values calculated with the counts below the detection limit of quantification are marked with an asterisk; (**a**) 8.0 × 10^3^ CFU/mL; (**b**) 8.0 × 10^5^ CFU/mL.

Figure 5 shows the voltage and current waveforms observed when an impulse voltage with a pulse width of 6.9 µs was applied. In the voltage waveform, the buildup and decay times were both approximately 4 µs. The current waveform almost exactly matched the voltage waveform. Figure 6 shows the sterilization rates when the application time was 10 min. Herein, the sterilization rate increased with increasing application time. In addition, there was an increase in the sterilization rate, with a threshold observed between 4.5 kV and 1.5 kV. For a 10 min application time, the decrease in cell count was 1.1 log at a bacterial density of 8.0 × 10^3^ CFU/mL and 4.4 log at a bacterial density of 8.0 × 10^5^ CFU/mL. At an application voltage of 1.5 kV, the sterilization rate did not increase above a fixed value, regardless of the application time. No detectable alteration in the count of viable bacteria was observed in the absence of applied voltage, and the rate of sterilization was almost zero. There was no difference in the bacterial sterilization rates obtained with different initial concentrations, and the increase in the bactericidal rate was the same for the initial concentrations of 8.0 × 10^3^ CFU/mL and 8.0 × 10^5^ CFU/mL. The change in pH at all voltages was measured, and almost no change was observed from 6.2.

### 3.3. Sterilization by Impulse Voltage with Rapid Buildup

**Figure 7:**
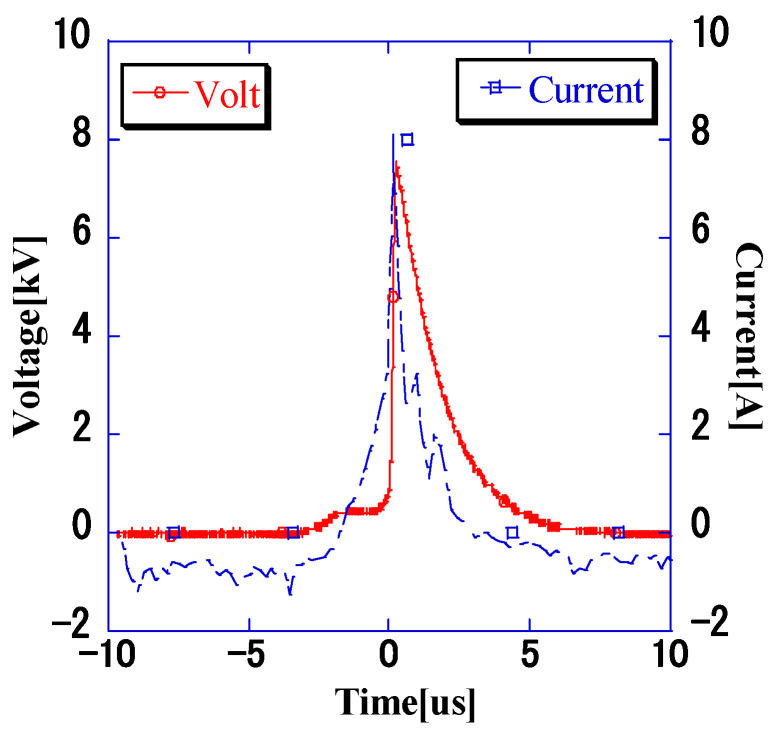
Voltage and current waveforms at a pulse width of 1.7 μs. The shape of the voltage and current waveforms are similar.

Next, the circuitry of the impulse voltage generator was changed to apply impulse voltages with rapid buildup to the *VRE*. The corresponding voltage and current waveforms are shown in Figure 7. The voltage waveform buildup time was 0.2 µs, and the decay time was 4 µs. A comparison with the results presented in Section 3.2 revealed that the decay time was the same; however, the buildup time was considerably shorter. The current waveform had the same shape as that of the voltage waveform, as indicated in Section 3.2.

**Figure 8:**
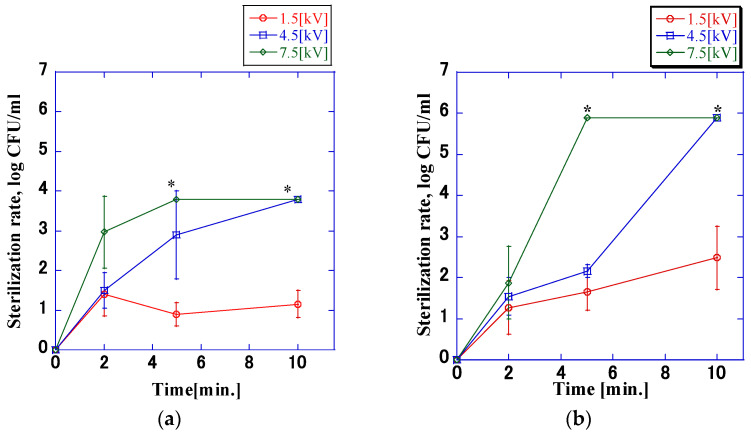
*VRE* sterilization rate at impulse voltage application times of up to 10 min (pulse width: 1.7 μs). There was a large difference between the sterilization rates at applied voltages of 4.5 kV and 1.5 kV. In particular, it could not be completely inactivated when the applied voltage was 1.5 kV. This was similar to the result when the impulse voltage with a pulse width of 6.9 µs was applied. Values calculated with the counts below the detection limit of quantification are marked with an asterisk; (**a**) 8.0 × 10^3^ CFU/mL; (**b**) 8.0 × 10^5^ CFU/mL.

Figure 8 shows the sterilization rate when different impulse voltages were applied to the different cell densities. The higher the application voltage was, the higher the sterilization rate. There was a large difference between the sterilization rates achieved with application voltages of 7.5 kV and 4.5 kV compared to those achieved with an application voltage of 1.5 kV. In particular, complete sterilization was not achieved at an application voltage of 1.5 kV. Additionally, the pH of the bacterial suspension did not change significantly at any voltage. These results were consistent with those reported in Section 3.2.

### 3.4. Temperature Change in the Bacterial Suspension When Impulse Voltage Was Applied

**Figure 9:**
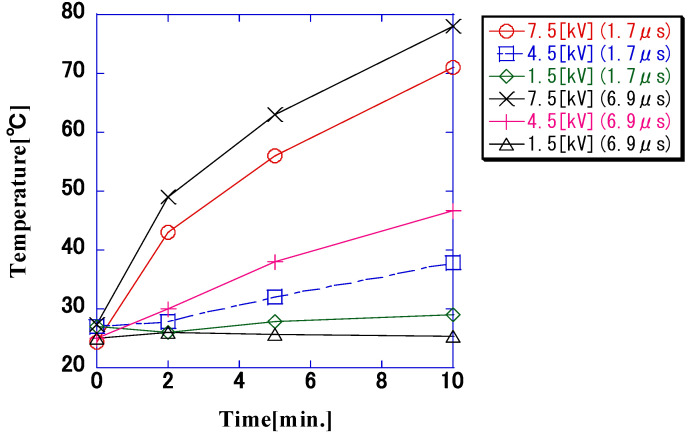
Temperature changes observed in the bacterial suspension (8.0 × 10^5^ CFU/mL) due to impulse voltage application. The temperature of the bacterial suspension increased with increases in the applied voltage and pulse width. At a voltage of 7.5 kV and a pulse width of 6.9 μs, the bacterial suspension reached a maximum temperature of 78.5 °C.

The temperature changes observed in the 8.0 × 10^5^-CFU/mL bacterial suspension when each impulse voltage was applied are shown in Figure 9. The temperature of the bacterial suspension increased with the application time, and the temperature increased with the increase in the applied voltage and pulse width. Ten minutes after applying the impulse voltage, the temperature exceeded 70 °C at an applied voltage of 7.5 kV, whereas it was approximately 40 °C at an applied voltage of 4.5 kV. The temperature increase was greater with a longer pulse width, even at the same voltage.

### 3.5. Sterilization by Impulse Voltage with Temperature Control of the Bacterial Suspension

**Figure 10:**
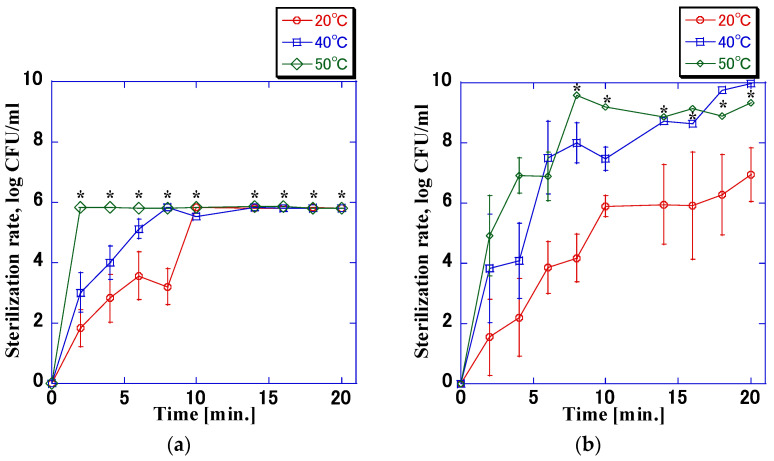
Variation in the sterilization rate when the temperature of the bacterial suspension was controlled (7.5 kV, 6.9 μs, 200 Hz). The sterilization rate increased with the increase in temperature. When the bacterial suspension was at 40 °C and 50 °C, the sterilization rate increased significantly compared with that at 20 °C, and the rate was >9.0 log. Values calculated with the counts below the detection limit of quantification are marked with an asterisk; (**a**) 8.0 × 10^5^ CFU/mL; (**b**) 8.0 × 10^9^ CFU/mL.

Figure 10 shows the change in the sterilization rate when the impulse voltage was applied and the temperature of the bacterial suspension was controlled. There was no difference in the change in bactericidal activity when the bacterial concentration was set at 8.0 × 10^5^ CFU/mL compared to 8.0 × 10^9^ CFU/mL, considering the upper limit of sterilization rate. For bacterial suspension temperatures of 50 °C and 40 °C, the sterilization rate at 50 °C was slightly higher than that at 40 °C. At both temperatures, the maximum sterilization rate was achieved when the application time was approximately 8–10 min. At 20 °C, the sterilization rate was lower than that at 50 °C. Even with an application time of 20 min, the sterilization rate was 6.9 log, which did not reach the maximum sterilization rate of 9.0 log. No increase in the sterilization rate was observed at any temperature when no impulse voltage was applied.

### 3.6. Change in the Conductivity of the Bacterial Suspension by the Application of Impulse Voltage

When bacteria were suspended in sterilized ultrapure water and the conductivity was measured, the conductivity was found to be <0.01 μs/cm at temperatures up to 50 °C. This was the detection limit of the instrument, and no change was observed.

**Figure 11:**
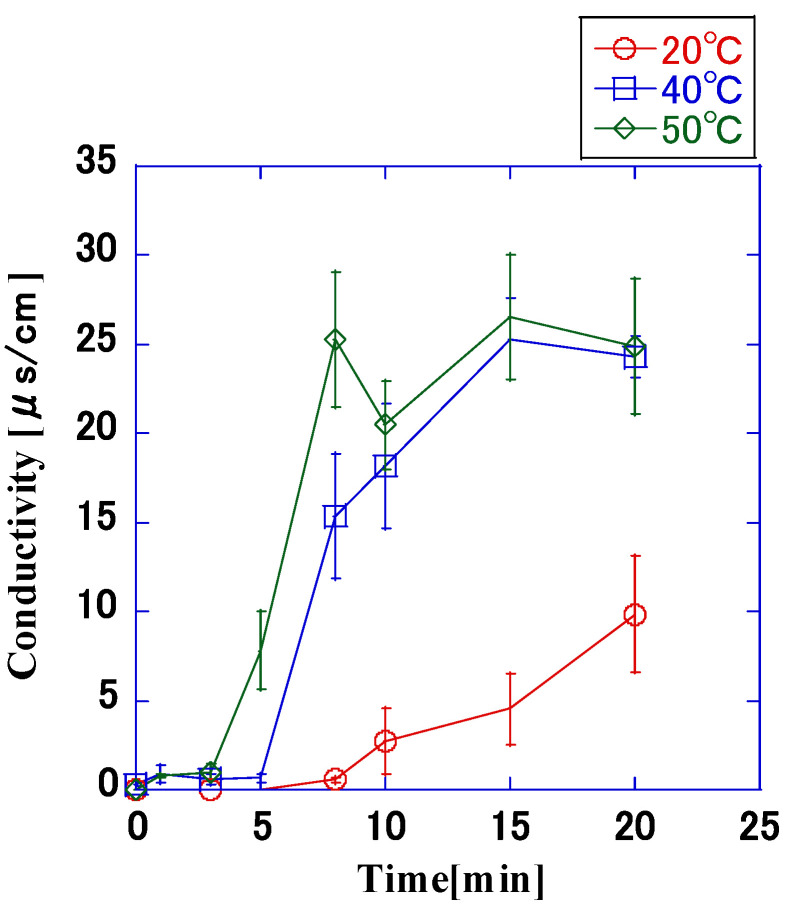
Variation in the electrical conductivity when the temperature of the bacterial suspension was controlled (8.0 × 10^9^ CFU/mL, 7.5 kV, 6.9 μs, 200 Hz). The conductivity increased as the temperature increased. Due to the detection limit of the conductivity meter, conductivity <0.01 μs/cm could not be measured, and the conductivity was constant. There are no error bars at the detection limit.

Figure 11 shows the change in conductivity when impulse voltage was applied for different application times. When the temperature of the bacterial suspension was 40 °C, the conductivity increased rapidly until the application time of 8 min and then continued to increase with increasing application time, gradually. When the application time was 20 min, the conductivity was approximately 25 μs/cm. When the temperature of the bacterial suspension was 20 °C, conductivity increased after 10 min of application; however, the rate of increase was smaller than that at 40 °C. At this time, the conductivity was approximately 10 μs/cm. When the temperature of the bacterial suspension was 50 °C, the change in conductivity was larger than that at 40 °C, and the conductivity was approximately 25 μs/cm at 8 min application time.

### 3.7. Change in Electrical Conductivity with Sterilization Rate

**Figure 12:**
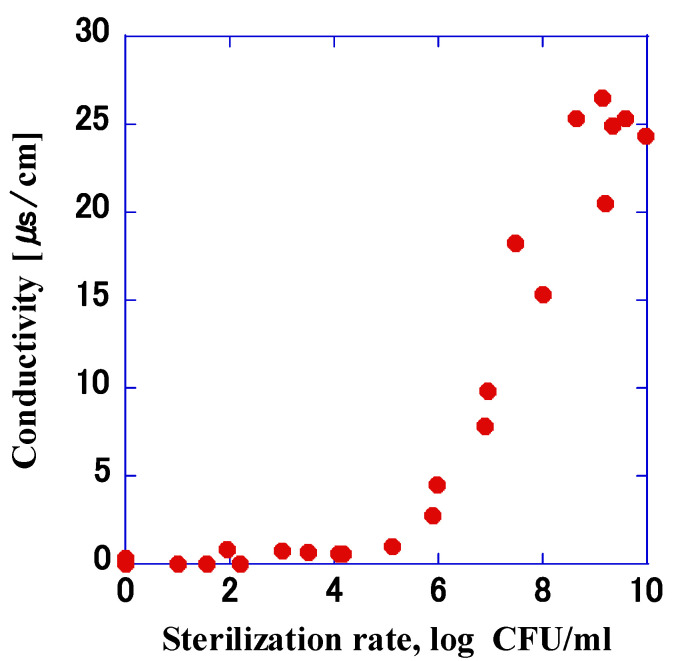
Relationship between the rate of sterilization and electrical conductivity obtained by impulse voltage application. There was a correlation between the conductivity and the rate of sterilization for conductivity >0.01 μs/cm (*p* > 0.05).

The relationship of the sterilization rate with the conductivity obtained in Section 3.5 and Section 3.6 was analyzed (Figure 12). For the number of bacteria > 6.0 log, the conductivity increased as the rate of sterilization increased (*p* > 0.05). However, for the number of bacteria < 6.0 log, the conductivity was constant at 0.01 μs/cm, which was the detection limit of the conductivity meter.

## 4. Discussion

Considering the impedance of the bacterial suspension, the voltage and current waveforms match the waveforms shown in Figure 5 and Figure 8, and the bacterial suspension has a majority of real (resistance) components. Baba et al. analyzed the electric field distribution at a frequency of 2 MHz for a liquid with higher conductivity and found that the electric field distribution is independent of the dielectric constant and is determined only by the conductivity [18]. The pulse waveform applied in this experiment is in the order of μs (a few kHz), which indicates that the target bacterial liquid is resistive.

Figure 4 and Figure 6 show that there was no difference in the sterilization rate at voltages of 7.5 kV and 1.5 kV, but the difference at 4.5 kV was significant. With a pulse width of 1.7 μs, the sterilization rate reached 6.0 log within 10 min of application, whereas with a pulse width of 6.9 μs, the sterilization rate reached 6.0 log within 5 min. Gintautas et al. and Frey et al. stated that pulse widths that differ by ≥100 times have an impact on the sterilization rate. The comparison revealed that the pulse widths of the voltage waveforms used in this experiment differed only by approximately four times, which was small enough to ensure that no difference in the sterilization rate is expected [19,20,21,22,23,24]. However, when comparing the time required for the temperature of the bacterial suspension to reach 38 °C at a voltage of 4.5 kV, it was found that application times of 10 and 5 min were required at a pulse width of 1.7 μs and 6.9 μs, respectively. The rate of increase in temperature was approximately double, which is consistent with the rate of increase in the sterilization rate. It is assumed that this is the reason for the difference in sterilization effects. To understand the relationship between sterilization rate and electrical parameters at a bacterial concentration of 8.0 × 10^5^ CFU/mL, multiple regression analysis was performed. The results are shown in Table 1. The disinfection rate did not depend on the pulse width, and there was no significant difference (*p* > 0.05). The correlation between the applied voltage and the application time was confirmed (*p* < 0.05), and the correlation between the applied voltage and the application time was higher than that between the applied voltage and the application time.

The change in the temperature of the bacterial suspension caused by impulse voltage is an important factor for determining the effectiveness of sterilization rate [25,26]. If the pores created in the cell membrane by the pulsed electric field are larger than a certain size, the intracellular fluid will flow out and the bacteria will die. The state of the phospholipids that make up the cell membrane is temperature dependent. Furthermore, a higher temperature is associated with a weaker lipid aggregation state; consequently, the sterilization treatment becomes more effective [25,26]. Heat is reported to be a factor that inhibits the repair of pores in the cell membrane, which may further enhance the sterilizing effect produced by impulse voltage [27,28]. Changes in the sterilization rate for the bacterial suspension at a constant temperature demonstrate that (Figure 10) a higher temperature of the bacterial suspension would lead to a higher sterilization rate. It has been reported that *E. faecium* could not maintain gene expression at temperatures above 60 °C [15]. In this experiment, the bacteria were inactivated even when the temperature of the bacterial suspension was <50 °C, which indicated that the death of the bacteria was not only caused by heat but also by the electric field. To understand the relationship between the temperature of the bacterial suspension, electrical parameters, and sterilization rate, we performed a multiple regression analysis based on the results of Figure 10b as before. The results are shown in Table 2. The temperature of the bacterial diluent and the application time were correlated with the sterilization rate (*p* < 0.05). The correlation of the bacterial diluent with the temperature was higher than that with the applied voltage. This result is consistent with the above discussion of electrical parameters and disinfection rate.

Longer application times lead to proportional increases in the conductivity; furthermore, the higher the temperature of the bacterial suspension was, the higher the conductivity. When the temperature of the bacterial suspension was 40 °C and 50 °C, the rate of increase in conductivity was similar, and at 20 °C, the conductivity was lower than that at 40 °C. This change in conductivity was similar to the change in the sterilization rate shown in Figure 10. Therefore, we analyzed the relationship between the sterilization rate and conductivity obtained in the previous experiment, and as shown in Figure 12, there was a correlation between the sterilization rate and conductivity when the number of bacteria was ≥6.0 log. When a pore opens in the cell membrane due to the application of pulsed high voltage, intracellular fluid flows out. Through experiments, Gintautas et al. showed that the concentration of potassium ions leaked from the cell increases with increasing electric field strength that can perforate the cell membrane [20]. In the present experiment, considering that the extracellular area was ultrapure water and the ion concentration was extremely low, the impulse voltage caused intracellular fluid ions to flow out of the cell, which resulted in a higher ion concentration and an increased conductivity. In addition, given that the conductivity increased with the rate of sterilization when the number of bacteria was ≥6.0 log, it may be possible to use the measure of conductivity as a means to rapidly estimate the number of bacteria that were sterilized by the pulsed high voltage. In order to determine the relationship between conductivities >0.01 μS/cm, which is the detection limit of the conductivity meter, and sterilization rate, regression analysis was performed, and the regression curve was derived as follows:

Y = −3.31X − 9.68(3)

where X indicates sterilization rate and Y indicates conductivity.

Table 3 shows that there is a correlation between conductivity and sterilization rate (*p* < 0.05). Since the intercept of the regression curve is negative, it may not be linearly proportional. In this experiment, it was difficult to measure conductivities <0.01 μs/cm due to the detection limit of the conductivity meter; however, by measuring lower conductivity, it is possible to derive the sterilization rate when the number of bacteria is <6.0 log.

Previous studies have only applied pulse voltages to *VRE*, without investigating the bactericidal effect of voltage waveform (pulse width), which is difficult to vary. While previous experiments mentioned the temperature of the bacterial solution, they did not control for temperature. The present experiments have clarified these conditions, and multiple regression analysis has highlighted the importance of temperature increase due to voltage application.

Furthermore, this sterilization method, which causes intracellular fluid to flow out, was found to allow for estimating the rate of sterilizations by changes in conductivity, a novel finding. Conventional impedance measurement methods using electrical means require collecting minute amounts of bacterial fluid (less than 1 mL) and detecting it using a high-frequency power supply, making them neither simple nor inexpensive [29,30,31,32]. However, measuring conductivity may allow for quickly and inexpensively estimating the number of viable bacteria, as demonstrated in this experiment. Although this method is limited to pulse application, the value obtained could be used to control the output voltage by providing feedback to the impulse power supply, enabling a highly energy-efficient sterilization method.

## 5. Conclusions

In this study, we tried to sterilize *VRE* using impulse voltage. We developed an impulse power supply and measured the sterilization rate by changing the voltage, pulse width, and temperature of the bacterial suspension. In addition, we measured the changes in electrical conductivity with the sterilization rate. The results are as follows:

1. Two types of impulse voltages with different buildup times were applied to *VRE*. The voltage waveforms and the current waveforms were almost identical, which indicated that bacterial suspension has a resistance load.
2. The rate of *VRE* sterilization differed depending on the value of the impulse voltage applied. When the application voltage was 7.5 kV or 4.5 kV, complete sterilization of the bacteria was observed at longer application times.
3. Impulse voltage was applied while controlling the temperature of the bacterial suspension. The higher the temperature of the bacterial suspension was, the higher the sterilization effect of the impulse voltage, and a sterilization rate of >9.0 log was achieved.
4. We confirmed the change in the conductivity of the bacterial suspension using impulse voltage. Our results suggested the presence of a correlation between the sterilization rate and conductivity.

This research clarified the sterilizing effect of the voltage waveform, specifically the pulse width, and the temperature of the bacterial solution on drug-resistant bacteria. In addition, in the context of this sterilization method, where intracellular fluid is shed, a new discovery is the potential to estimate the number of bacteria sterilized by tracking changes in conductivity. Although limited to pulsed disinfection methods, this change in conductivity may facilitate rapid and cost-effective identification of viable bacteria.

The ultimate goal of this study is to disinfect against drug-resistant bacteria in wastewater. Future research needs to confirm gene inactivation and bactericidal effects in bacterial solutions with high electrical conductivity.

## Figures and Tables

**Table 1:** Multiple regression analysis between *VRE* sterilization rates and electrical parameters: part I.

	Coefficients	t Stat	*p*-Value
Intercept	−2.34 × 10^5^	−1.95	6.26 × 10^−2^
Application time	2.82 × 10^4^	2.34	2.76 × 10^−2^
Voltage	4.73 × 10^4^	3.08	5.00 × 10^−3^
Pulse width	−2.97 × 10^3^	−1.68 × 10^−1^	8.68 × 10^−1^

**Table 2:** Multiple regression analysis between *VRE* sterilization rates and electrical parameters: part II.

	Coefficients	t Stat	*p*-Value
Intercept	−1.1 × 10^9^	−3.89	1.80 × 10^−4^
Application time	4.40 × 10^7^	2.76	6.99 × 10^−3^
Temperature	2.88 × 10^7^	5.52	2.75 × 10^−7^

**Table 3:** Multiple regression analysis between *VRE* sterilization rates and electrical conductivity.

	Coefficients	t Stat	*p*-Value
Intercept	−9.68	−3.48	2.86 × 10^−3^
Sterilization rate	3.31	8.17	2.74 × 10^−7^

## Data Availability

Not applicable.
